# Promoting Mathematics Learning in Young Children Through the Use of Embodied Mathematics Teaching Modules

**DOI:** 10.3390/bs16060875

**Published:** 2026-06-01

**Authors:** Yin-Yin Chen, Su-Chiao Wu, Yu-Liang Chang, Lancelote Andy Chang

**Affiliations:** 1 Department of Electrical and Mechanical Technology, National Changhua University of Education, Changhua 500207, Taiwan; yinyin35@yahoo.com.tw; 2 Department of Early Childhood Education, National Chiayi University, Chiayi 621302, Taiwan; angelwu@mail.ncyu.edu.tw; 3 Department of Education, National Chiayi University, Chiayi 621302, Taiwan; 4 College of Natural Resources, University of Idaho, Moscow, ID 83843, USA; chan3344@vandals.uidaho.edu

**Keywords:** embodied/embodiment, embodied mathematics teaching modules, mathematics learning, young children

## Abstract

The “theory of embodied mathematics cognition for young children” is based on the use of the senses and body movement to perceive reality and, in turn, produce different perceptions and concepts. Given the importance of embodied mathematics learning for young children and the lack of relevant research, using embodied mathematics teaching modules to promote children’s understanding of mathematics at the kindergarten level is essential. Accordingly, the main purpose of this study was to enhance young children’s mathematics learning by implementing designated embodied mathematics teaching modules as an intervention. A quasi-experimental design with pre- and post-tests was employed, using the “Taiwanese Embodied Mathematics Assessment—Short Form (TEMA-SF)” to collect data through clinical interviews. The data were then analyzed quantitatively, along with exploratory descriptions. The findings and discussions of this study are reported in two major sections: First, we reveal and discuss how the revised version of the 10 embodied mathematics teaching modules, as the intervention for the experimental group, significantly promoted the children’s embodied mathematics learning in overall scores and geometry. Then, since the TEMA-SF was administered through clinical interviews, we explore how the TEMA-SF can serve as a comprehensive yet developmentally appropriate assessment tool for measuring young children’s mathematics learning performance.

## 1. Introduction

### 1.1. The Importance of Early Mathematics Knowledge

Prior achievement in mathematics is a strong predictor of later success in the subject (Bodovski & Youn, 2011). Stevenson and Newman (1986) reported that mathematical knowledge acquired during early childhood is moderately correlated with later mathematics achievement in the 10th grade, and Watts et al. (2014) also indicated that early mathematics skills continue to predict subsequent achievement up to the age of 15, even after accounting for factors such as early reading ability, cognitive skills, and family and child traits. Early number sense decisively shapes children’s trajectory in mastering mathematics (Jordan & Devlin, 2023); for example, mental thinking and using the fingers to count are vital to the development of early number sense (Krenger & Thevenot, 2026). Foster et al. (2015) indicated that prior knowledge plays a crucial role in learning and future understanding across many topics and skills; however, the influence of early mathematics knowledge is particularly powerful and long-lasting. Moreover, children who begin with stronger mathematics skills tend to experience more rapid growth in their mathematical abilities compared to peers with lower initial skills (Aunola et al., 2004; Duncan et al., 2007). Importantly, this finding holds true even when controlling for children’s early cognitive abilities, behavior, and other key background variables (Romano et al., 2010).

### 1.2. Embodied Mathematics Cognition and Learning for Young Children

In recent years, embodied mathematics teaching has emerged as a significant area of interest in mathematics education, drawing on post-cognitive theory. Scholarship has shifted away from merely observable learning outcomes, instead prioritizing investigating how students interact with their environment and how they construct cognitive learning processes through their body and mind. The idea of “embodied mathematics cognition” in early childhood education is based on the premise that learners develop perceptual concepts through engagement with external stimuli via their senses and bodily actions (Glenberg, 2010). Experts suggest that all mathematical learning is fundamentally grounded in this initial, embodied understanding of the world (Abrahamson, 2017; Abrahamson & Sánchez-García, 2016; Hutto et al., 2015) rather than confined to abstract mental operations (Barsalou, 2008; Wilson, 2002).

When applied to mathematics education, it is argued that learners can achieve deeper mathematical understanding when abstract concepts are grounded in physical activity (Abrahamson & Sánchez-García, 2016; Mavilidi et al., 2022). Multimodal learning is a central component of this approach, emphasizing the importance of visual, auditory, kinesthetic, and tactile modalities in the learning process. By integrating gesture, whole-body movement, and hands-on manipulation, embodied teaching provides diverse entry points for learners to engage with mathematical ideas (Alibali & Nathan, 2012).

In the context of early childhood, embodied approaches are especially relevant. Young children, who are typically in the preoperational stage of development (Piaget, 1952), rely heavily on physical interaction and sensory input to understand their environment. Recent research has increasingly highlighted that young children’s early mathematical intuitions—such as number, space, and shape intuitions—emerge through physical activity and everyday experiences (Sarama & Clements, 2009). Embodied learning builds upon this premise, emphasizing the transformation of informal, bodily-based experiences into formal mathematical concepts through structured, physically grounded learning opportunities. van’t Noordende et al. (2017) demonstrated that young children’s number–space mapping is influenced by physical interactions such as reaching and hand preference, indicating that numerical cognition is fundamentally expressed. Movement and gesture, therefore, play a central role in the development of numerical understanding.

Spatial reasoning, another core component of early mathematics, also benefits significantly from embodied experiences. Naylor and Cowan (2023) reviewed evidence indicating that kinesthetic activities—such as walking along number lines or manipulating objects—enhance children’s comprehension of spatial concepts, including position, orientation, and distance. Importantly, embodied learning extends beyond individual movement to include material and social interactions. Lenz Taguchi et al. (2022) found that young children in kindergarten settings use bodily positioning and engagement with physical materials to participate meaningfully in mathematical activities. Their findings emphasize that mathematical learning is not only cognitive but also relational and situated within the physical and social environment.

Gestures also serve as vital tools for externalizing thought processes during mathematical problem-solving. Amit and Fried (2021) showed that kindergarten children use gestures and physical object manipulation to construct and communicate mathematical ideas, enabling complex reasoning even in the absence of fully developed verbal or symbolic language. Finally, the practical implications of embodied learning are underscored by Fischer et al. (2011), who demonstrated that embodied training—such as the use of finger–numeral configurations—enhances arithmetic performance in young children. These findings support the systematic integration of embodied strategies in early childhood mathematics curricula to support the development of both numerical and spatial competencies.

### 1.3. Embodied Mathematics Learning Task Design

Abrahamson (2024) argued that learning a mathematical concept involves developing a new perceptual capacity by solving a motor control problem. Creating a learning environment enhances one’s “perception-for-action” through the attentional anchor, where, for example, physical or spatial markers guide children as they connect movement to abstract ideas or concepts (Abrahamson & Lindgren, 2014). In this learning process, “the attentional anchor serves as a percept-to-concept pivot—from doing to thinking-about-doing” (Abrahamson, 2024, p. 149). Based on this argument, mathematics learning activities for young children should go beyond basic movement and focus on “mindful movement” designed to achieve specific mathematical objectives (Shoval, 2011). One of the key principles that needs to be considered when designing embodied mathematics learning tasks is mathematical congruency; i.e., the movement should clearly illustrate the mathematical concept being taught (Björklund et al., 2020). For example, using fingers to jump forward on a number line to show addition works well since the movement aligns with the idea that numbers get bigger (Nathan & Walkington, 2017). Leaning task design should evolve from hands-on experience to more abstract thinking, e.g., starting with physical activity, then using gestures or digital tools to convey ideas, and finally leading to formal mathematical symbols (Way & Ginns, 2024). Moreover, many teachers often treat physical activity as merely regular exercise or playtime rather than a critical component of teaching mathematics, underscoring the need for more teacher training (Paniagua & Istance, 2018). In short, developing embodied mathematics learning tasks that take advantage of young children’s natural tendency to move can help them think about how their body movements relate to the specific mathematical concepts they are learning (Way & Cartwright, 2025). However, less research has focused on the design and implementation of embodied mathematics teaching and learning in early childhood education in Taiwan. In order to make up for this research gap, the research team proposed and executed two research projects starting in the 2018~2019 academic year, in which professional development (PD) programs for in-service kindergarten teachers were designed and implemented to furnish them with knowledge and skills essential for embodied mathematics teaching and learning (Chang et al., 2024; Wu & Chang, 2022, 2025).

### 1.4. Embodied Mathematics Learning Assessment

Assessing young children’s mathematical understanding requires developmentally appropriate and culturally responsive strategies that recognize the distinctive ways through which young children express their ideas. In contrast to older children, young children often present their mathematical thoughts through play, gestures, manipulation of concrete objects, and informal language (Clements & Sarama, 2021; Sarama & Clements, 2009). Therefore, for young children, mathematics learning assessments should incorporate observational, performance-based, and interactive approaches rather than traditional assessment tools (e.g., paper-and-pencil tests). Effective assessment for young children aligns with cognitive, emotional, and motor development. Consequently, assessments should emphasize contextual tasks involving tangible materials, hands-on manipulation, and opportunities for verbal explanation and gestural communication (Ginsburg et al., 2008). Furthermore, early mathematics learning assessment should be conducted formatively, providing continuous insights into conceptual understanding and guiding instructional decisions. Authentic assessments implemented interactively, such as play or classroom dialog, enable more accurate representations of children’s knowledge and capabilities (NAEYC & NCTM, 2010).

There are various assessment strategies recommended for assessing young children’s mathematical understanding and performance: (1). Observation and documentation: Young children are observed during play and designated learning activities, and their behavioral actions when using number, shape, size, pattern, and spatial reasoning are recorded (Charlesworth, 2005). Anecdotal records, checklists, and learning stories are commonly used to monitor their long-term development. (2). Performance-based tasks: Mathematics learning activities are designed to employ diverse teaching aids or manipulatives, such as blocks, counting objects, or puzzles, allowing young children to manifest their understanding through action or movement. These tasks contextually assess targeted mathematical concepts such as one-to-one correspondence, counting principles, subitizing, and measurement (Clements & Sarama, 2007). (3). Interviews and thinking aloud: Structured interviews and informal conversations provide interactive opportunities for young children to clearly clarify their ideas or reasons. Probing questions help educators identify misconceptions or emerging understandings (Ginsburg, 2009). (4). Gamified assessment: Designing and implementing gamified assessment tools that can be merged into learning activities could reduce young children’s anxiety and enable them to express their authentic thoughts (Gelman & Brenneman, 2004). Digital game-based tools are increasingly used to obtain real-time performance data within playful contexts.

Additionally, several remarkable research-based mathematics assessment tools and frameworks have been developed and used to assess young children’s mathematics learning, e.g., the Test of Early Mathematics Ability–Third Edition, the Woodcock–Johnson III Tests of Achievement, the Child Math Assessment, and the Tools for Early Assessment in Mathematics (Purpura & Lonigan, 2015). However, these measures are generally limited in both content and sensitivity in discovering potential differences in early mathematics development among young children (Clements et al., 2008; Weiland et al., 2012). In fact, Clements et al. (2008) developed the “research-based early mathematics assessment (REMA)” to address the abovementioned gaps. Based on empirical evidence on learning trajectories in mathematics, the REMA assesses a broader range of early mathematics skills than more widely used measures (Clements, 2007). The learning trajectories, defined as “descriptions of children’s thinking and learning in a specific mathematical domain, …, created with the intent of supporting children’s achievement of specific goals in that mathematical domain” (Clements & Sarama, 2004, p. 83), summarize typical developmental progressions in mathematical thinking that support mathematical instruction and assessment (Sarama & Clements, 2009).

Later, because the comprehensive nature (125 items in total) of the REMA might limit its use, Weiland et al. (2012) validated a 19-item short-form version of the full 125-item REMA (Clements et al., 2008) titled “REMA-SF”, which includes items only for the earliest learning trajectories related to mathematical development that can be used to assess pre-kindergarten (Pre-K, 4-year-old) and kindergarten (5-year-old) children’s numeracy and geometry skills. Importantly, no research has examined how REMA and REMA-SF can be used to assess young children’s mathematics learning performance in Taiwan, nor their use in practical settings. In addition, due to potential differences in children’s cultural and developmental backgrounds, as well as the teaching and learning environment in practical settings, REMA and REMA-SF need to be thoughtfully revised to better align with the Taiwanese context for further examination. Therefore, to address this gap, this study was conducted as a follow-up to the previous two research projects during the 2020~2021 academic year.

### 1.5. The Current Study

#### 1.5.1. Linking Theoretical Framework and Research Gap to the Current Study

Analysis of the literature on embodied mathematics teaching for young children reveals that it is deeply rooted in bodily action and perceptual experience rather than defining mathematical learning as abstract and symbolic. This perspective emphasizes that mathematical concepts can be understood through the dynamic interaction of movement, sensation, and cognition. To support the transition from action to abstraction, instruction such as spatial markers, gestures, or manipulatives, which are called attentional anchors, may be used. Furthermore, embodied mathematics teaching is inherently multimodal, integrating whole-body movement, multisensory strategies, visual representations, and tactile interaction. Instruction emphasizes a concrete–representational–abstract progression to develop sequencing skills from gestures to drawings and, ultimately, formal symbolic notation. To ensure that abstract reasoning is grounded in meaningful experience, it is important to sustain engagement and align with young children’s diverse developmental characteristics. Notably, embodied mathematics teaching is play-based and contextualized within meaningful and interactive scenarios that draw on children’s natural experiences while promoting deeper conceptual learning. Accordingly, the design of the teaching module and the revision of the assessment of this study were synthesized from the principles above.

#### 1.5.2. Research Purposes and Questions

Since mathematical learning for young children is complex and unique to each child, understanding how children learn mathematics and how to design appropriate mathematical learning activities at the kindergarten level is vital. The “theory of embodied mathematics cognition for young children” is based on the use of body senses and movement to perceive reality and, in turn, to produce different perceptions and concepts. Thus, using embodied mathematics teaching modules to promote their understanding of mathematics is the main task for achieving the above-mentioned goals. Based on this argument, two research projects were conducted during the academic year in the 2018~2020 period, in which PD programs were provided to enhance the professional growth of designated kindergarten teachers in designing and implementing embodied mathematics teaching and learning techniques. As a result, a sustainable task design PD model was generated for the current study (Chang et al., 2024, p. 12). A total of 10 embodied mathematics teaching modules were also designed and implemented for promoting young children’s learning of mathematics (Chang et al., 2024; Wu & Chang, 2022, 2025). Accordingly, the aim of this study was to examine the effectiveness of implementing the designed embodied mathematics teaching modules for young children’s mathematics learning. A quasi-experimental design with pre- and post-tests was employed with an embodied mathematics learning assessment tool, including two groups of young children: an experimental group and a control group. An intervention using the revised embodied mathematics teaching modules was implemented in the experimental group, while the control group used a traditional teaching approach. The research questions and hypotheses are posed as follows:

1. Are there statistically significant differences in the mathematics learning gains between the two groups of young children after the intervention?
  (1) Hypothesis I (between-group baseline comparison, pre-test):
    There will be no significant difference between the two groups of young children in their mathematics learning before the intervention.
  (2) Hypothesis II (between-group post-intervention analysis, post-test):
    There will be significant differences between the two groups of young children in their mathematics learning after the intervention.
  (3) Hypothesis III (within-group gains, experimental intervention implementation):
    There will be significant gains in mathematics learning among young children in the experimental group after the intervention.
2. For young children in the experimental group, what are the exploratory descriptions of young children’s mathematics learning, as evidenced by changes in their performance levels on the embodied mathematics learning assessment from pre-test to post-test?

## 2. Materials and Methods

### 2.1. Methodology and Participants

A quasi-experimental design with pre- and post-tests was employed in this study. The participants of this study were four certified kindergarten teachers from two kindergartens in Southern Taiwan and their 5-year-old children. Kindergarten A (2 teachers and 25 children) was assigned as the experimental group, while Kindergarten B (2 teachers and 26 children) was the control group. A purposive sampling approach was used to select the two kindergartens (and their teachers), based on a survey using the “instrument of understanding and implementation of the theory of embodied mathematics learning cognition for young children” (Wu & Chang, 2022, p. 1). As a result, the teachers’ scores of the targeted kindergartens were approximately similar, indicating their basic understanding of embodied mathematics teaching and learning was approximately at the same level. All four teachers in Kindergarten A and B had more than 10 years of teaching experience and a bachelor’s degree, but none had experience implementing the embodied mathematics teaching modules. The two kindergartens were located in the urban area, where all children were typically developing Taiwanese with a middle socio-economic status. None of these young children had disabilities or experience of embodied mathematics learning.

In Kindergarten A, the two teachers in the experimental group participated in the PD program, using the sustainable task design model (Chang et al., 2024), to promote understanding, design, and implementation of the “theory of embodied mathematics cognition for young children” during the 2021~2022 academic year. Grounded in the thematic teaching plans in Kindergarten A, they modified the 10 embodied mathematics teaching modules developed in previous projects (Chang et al., 2024; Wu & Chang, 2022, 2025). Therefore, a revised version of 10 embodied mathematics teaching modules was used as the intervention to promote their young children’s embodied mathematics learning performance. The control group was taught using traditional teaching modules and learning activities that emphasized preparation for later formal learning at the elementary level, i.e., basic “reading, writing, and arithmetic” as the main learning content. Furthermore, a revised version of “the 19-item REMA-SF” modified by Weiland et al. (2012), named “Taiwanese Embodied Mathematics Assessment—Short Form (TEMA-SF)”, was employed to collect data in both the experimental and control groups. This embodied mathematics learning assessment tool was administered to both groups through clinical interviews as pre- and post-tests. The pre-test was administered at the beginning of the Spring 2022 semester, and the post-test was administered at the end of the same semester. Correspondent statistical analyses were employed (answering research question 1), along with an exploratory description (answering research question 2), to analyze the data corpus.

### 2.2. Experimental Embodied Mathematics Teaching Modules

#### 2.2.1. Previous Studies—Original Embodied Mathematics Teaching Modules

As previously mentioned, PD programs were provided to eight certified kindergarten teachers in a public kindergarten (Kindergarten P) in Southern Taiwan during 2018~2020 academic years (Chang et al., 2024; Wu & Chang, 2022, 2025). Jaworski’s (2006) teaching triad (i.e., management of learning, sensitivity to students, and mathematical challenge) served as the framework for the PD programs to furnish their understanding, design, and implementation of the “theory of embodied mathematics cognition for young children” by addressing the theoretical “why” of embodied learning and the practical “how” of classroom instruction in the first year. Four teachers with more than 10 years of teaching experience and a bachelor’s degree, selected from among the eight teachers in Kindergarten P as seed teachers in the second year, engaged in the professional dialog process to design “embodied mathematics teaching modules” (Chang et al., 2024). A task design PD model was employed in this dialog process, which integrated conceptual/theoretical foundations, students’ thinking, and three types of knowledge into ongoing task design discussions. In addition, the design process focused on merging newly developed embodied mathematics teaching and learning into the existing thematic teaching plans in Kindergarten P, and all embodied mathematics teaching modules were developed and refined through a cyclical process of planning and field-testing. (1) Pre-semester preparation: Before the school year began, researchers and teachers met to explore embodied design and develop initial teaching modules. (2) Field-testing experiment: During the semester, four teachers implemented these designs in their classrooms while being observed by the research team. (3) Feedback and revision: Weekly PD meetings (held on Wednesday afternoons) and post-class discussions were used to reflect on emerging issues and adjust the initial modules and learning activities based on their children’s actual responses. As a result, 10 embodied mathematics teaching modules were designed to promote young children’s embodied mathematics learning (Chang et al., 2024).

An “embodied design” approach (Abrahamson, 2009, 2014; Abrahamson & Shulman, 2019) was employed within the abovementioned PD process to ensure adequate prerequisite knowledge and capabilities for developing the embodied mathematics teaching modules. Abrahamson et al. (2020) claimed that embodied design is “a theory-to-practice approach to mathematics education” and “a pedagogical framework” that “draws on principles of genetic epistemology, Enactivism, ecological dynamics, and cultural-historical psychology to engage students’ naturalistic sensorimotor capacity and stage opportunities for guided negotiation between grounded ways of knowing and mathematical forms and practices” (p. 2). In fact, this embodied design approach is beneficial for teachers to assist young children’s mathematics learning.

Based on this theoretical framework, Wu and Chang (2025) generated two basic principles of “embodied mathematics curriculum and instructional design”:

1. Designing learning tasks reflecting real-life situations: Embodied mathematics learning tasks are designed and implemented as coherently as possible in young children’s daily lives in order to provide abundant opportunities for them to learn and perform in a real-life situation. For example, when teaching number concepts, the learning context is designed to emphasize representing, connecting, and manipulating integers. By providing concrete objects or teaching aids, young children can obtain more hands-on experience that connects them to real-world situations. This kind of embodied learning context also allows them to learn in a “perception–action cycle” (Tran et al., 2017), where these hands-on movements help produce changes in external (e.g., manipulatives) and internal (e.g., children’s interest or motive) environments that, in turn, affect later actions in a cyclical process. This cycle, which embraces the interaction among perception, environment, and behavioral action, is key to a child’s embodied learning (Chang et al., 2024).
2. Using pictures, images, manipulatives, and embodied operations to promote thinking: Instead of imparting young children with fixed knowledge, requiring them to memorize formulas, or having them perform technical calculations in a traditional mode, embodied mathematics teaching focuses on promoting their robust understanding of mathematical concepts by using multiple representations and providing diverse perspectives. These pedagogical practices allow young children to think and discuss during the “learning by doing” process and then solve real-life mathematical problems within the designated embodied learning tasks (Chang et al., 2024).
3. Several embodied mathematics teaching strategies, corresponding to the two basic principles, have been employed and were found to be effective in establishing active learning habits in the studied young children. For example, various inquiry and problem-solving tasks were designed to engage young children in an active, collaborative learning environment. These tasks also promote the interaction of the children’s previous and new experiences, which is essential for constructing a new mathematical concept and learning all concepts in unity. Furthermore, young children should be given ample time and a variety of learning materials (e.g., manipulatives, teaching aids) so they can explore the essence of mathematics. Many embodied mathematics learning tasks are designed so that young children can understand the relationships and connections among quantities by using embodied mathematical tools in a real-life context through, for example, counting, sorting, measuring, recording, and calculating. These embodied learning experiences allow “teachers and their children to act spontaneously, which, in turn, helps them to become active learners who can collaboratively interact with the environment and others” (Chang et al., 2024, p. 15).

Based on the theoretical framework of an “embodied design” approach and the two basic principles of “embodied mathematics curriculum and instructional design”, ten embodied mathematics teaching modules were developed for future use to enhance young children’s embodied mathematics learning. These ten modules were designed around two foci: (1). facilitating young children’s geometric and spatial discourse in the physical world and (2). fostering hands-on experiences that involve manipulating concrete objects to understand the relationships and connections of numbers and quantities. For instance, young children are encouraged to apply their understanding of geometric concepts (e.g., shape, orientation, and spatial relations) to describe patterns in daily life, as well as to recognize, name, and draw basic geometric shapes to interpret their environment and construct complex structures by using manipulatives. In addition, they are supported in developing number sense, including writing numerals and using numbers to solve “quantity” problems. This involves embodied tasks such as speaking quantities out loud, counting out specified amounts of the given objects, and understanding the total amount when combining two sets of quantities. Furthermore, these modules aim to stimulate the use of diverse counting strategies and subitizing and comparing quantities between two groups of objects. Finally, the design of the mathematical content and assessment tools across these ten modules is based on the Early Childhood Education and Care Curriculum Framework (Ministry of Education, Taiwan, 2016) and aligned with the Common Core State Standards for Mathematics (CCSSI, 2023) and the standards framework of the National Council of Teachers of Mathematics (NCTM, 2000). Table 1 and Table 2 present the main concepts of each module and how they are designed to align with the standards.

#### 2.2.2. Current Study—Revised Embodied Mathematics Teaching Modules

In this study, a revised version of 10 embodied mathematics teaching modules was developed (mainly in the Fall semester of 2021) and implemented as the intervention of the experimental group (in the Spring semester of 2022) in the selected class (containing 5-year-old children) taught by the two teachers. An example is shown (see Table 3 for details) of how the studied teachers adjusted the embodied mathematics teaching modules and implemented them as the intervention, as well as how they furnished the learning environment (e.g., learning centers) with various materials and manipulatives to promote their children’s mathematical understanding and skills. In this way, young children could learn the designated mathematical concepts in a real-life context, where they can explore and develop a better understanding and desirable skills. Moreover, the implementation of the revised embodied mathematics teaching modules in Kindergarten A was practically merged with the thematic teaching plans, emphasizing the relevance and consistency between the “thematic learning activities” and the “embodied modules”. Each revised module was basically designed to be taught in a week (5 days), with at least 2 h of embodied mathematics learning activities integrated into the regular thematic learning process.

**Figure 1:**
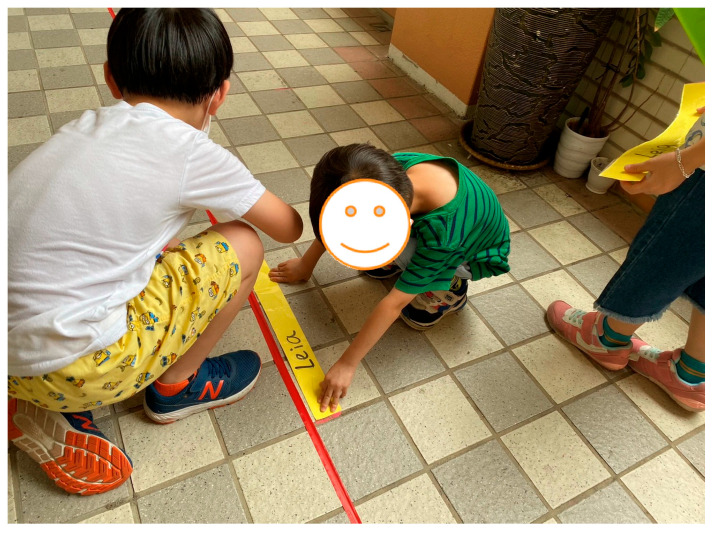
Children’s route planning for measuring the distance from the classroom to the office.

**Figure 2:**
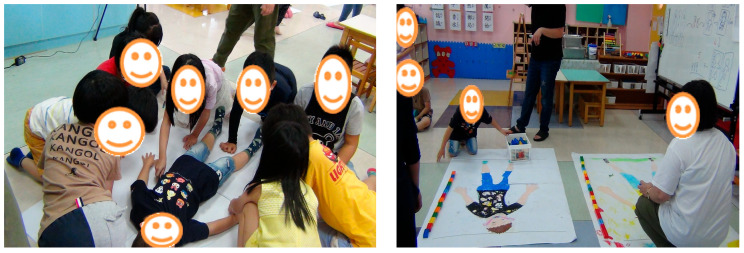
Children traced the body outlines of the two children lying on the floor.

In Kindergarten A, teachers adapted the measurement module (module 10) to their own thematic teaching and learning context, using the problem: “What is the distance from the classroom to the office?” (Figure 1). The children in the experimental group developed route-planning ideas and used different embodied tools to measure (e.g., hands, feet, blocks). In the end, they discovered that they needed to use one child’s foot consistently as a “foot ruler” to measure the required distance. After returning to the classroom, they compared the heights of two children. Through group discussion and task division, these children traced the body outlines of the two children lying on the floor. They then conducted both informal and formal measurements and concluded that measurement requires a “consistent unit” for comparison (Figure 2).

**Figure 3:**
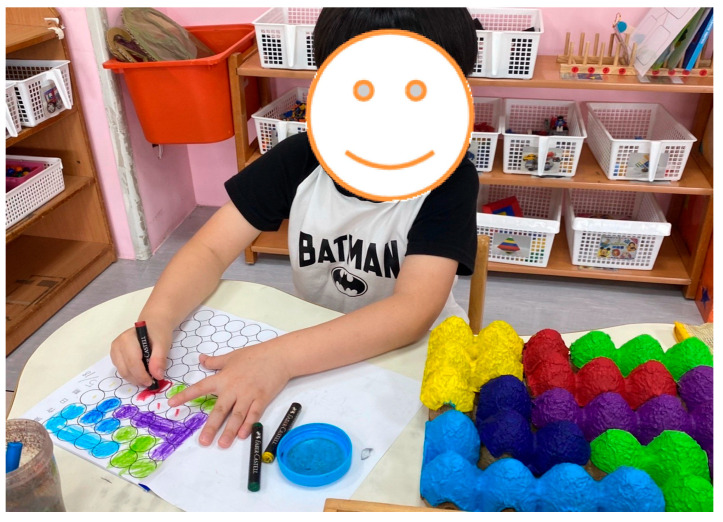
Self-made learning materials in the mathematics learning center.

**Figure 4:**
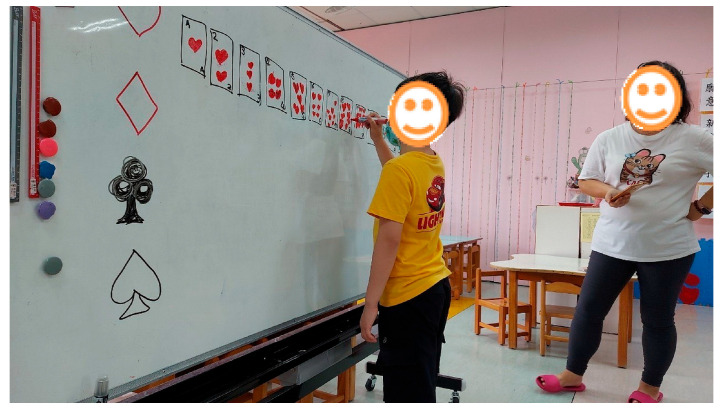
Playing card games—drawing cards on the board.

**Figure 5:**
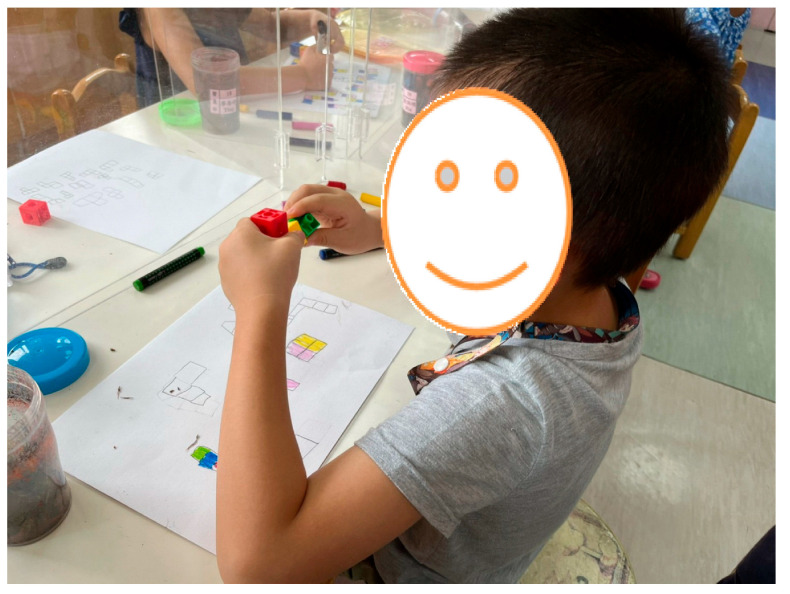
Activity of assembling four “unit blocks”.

In addition, in line with the revised version of the 10 embodied mathematics teaching modules, the teachers developed self-made learning materials for children to use in the mathematics learning center (Figure 3). One activity in this center involved children first observing the numbers, quantities, and arrangement order of playing cards, then designing their own playing cards, and finally playing card games (Figure 4). Another activity allowed children to assemble four “unit blocks” (Figure 5) to try to represent a three-dimensional structure on the learning sheet.

### 2.3. Embodied Mathematics Learning Assessment Tool

The “research-based early mathematics assessment (REMA)”, originally developed by Clements et al. (2008), is a diagnostic tool designed to assess children’s mathematical cognition and learning performance from the ages of 3 to 8 (see the original Table 1 in pp. 465–466 for example items). Unlike traditional assessments, the theoretical framework of the REMA is grounded in “learning trajectories (LTs)”—empirical paths that describe young children’s thinking progress as they learn mathematics from simple to complex concepts. This tool uses the Rasch model (Item Response Theory) to calibrate item difficulty to children’s ability, ensuring that each task corresponds to a specific developmental level along the LTs. Due to the comprehensive nature of the REMA (which contains 125 items), Weiland et al. (2012) validated a condensed version entitled the “REMA Short Form (REMS-SF)”, featuring only 19 items for the earliest learning trajectories related to mathematical development to assess the potential differences in kindergarten children’s numeracy and geometry skills without the limitations in both content and sensitivity. This REMA-SF focuses on assessing two core content domains—“numeracy” (e.g., verbal counting, object counting, number comparison, and compositions/decompositions) and “geometry” (e.g., shape recognition, composition of shapes, and spatial orientation)—and maintains high reliability and validity. Similar to the REMA, it also emphasizes how a child solves a problem through observing children’s behavioral actions (e.g., using verbal counting and other counting strategies such as “counting-all”, “counting-on”, or “skip-counting”), rather than just whether they provide the correct answer.

Items in the “REMA-SF” comprise two major parts, in which the item description, core competency, and level of thinking in the learning trajectory correspond to the perspectives of Sarama and Clements (2009) and are clearly described in the original Table 3 (Weiland et al., 2012, p. 317): (1). Number: counting (both verbal and object, 2 items), comparing number and sequencing (2 items), recognition of number and subitizing (4 items), composition of number (i.e., early arithmetic combinations, 2 items), basic numeral identification (1 item) and arithmetic addition (1 item). (2). Geometry: shape (at the earliest trajectory levels, 5 items), shape composition (1 item), and patterning at the lowest level (1 item). The REMA-SF is a psychometrically valid, theory-based assessment of young children’s early mathematics skills, with the Rasch model indicating adequate fit to its items. Consequently, the theoretical frameworks of the REMA (Clements et al., 2008) and the REMA-SF (Weiland et al., 2012) were employed in this study to design the embodied mathematics learning assessment tool.

Even though the REMA-SF is a valid assessment tool for examining young children’s mathematics skills (especially for numerical and geometric concepts and spatial reasoning), it needs to be carefully adjusted to the cultural context while being used in practical settings with diverse backgrounds (e.g., curriculum and instructional design, teacher professional training, and children’s capabilities). To achieve this purpose, the research team revised the REMA-SF via the following steps:

(1) Translating all items into traditional Chinese. The item description, core competency, and level of thinking in the learning trajectory of all 19 items were translated by the research team.
(2) Adding clear instructions to each item. In order to administer the assessment in a standardized manner, along with the scoring criteria, clear instructions were added to each item.
(3) Selecting appropriate concrete objects or teaching aids for each item. Concrete objects or teaching aids for each item were carefully chosen based on the targeted young children’s backgrounds and real-life experiences.
(4) Designing the scoring criteria for each item aligned with the core concept. According to the item description, core competency, and level of thinking in the learning trajectory of all items, a three-level rubric (10 points per item) was employed, where the score criteria for each item were added. The scoring process of the embodied mathematics assessment was administered through clinical interviews, in which young children’s behaviors were observed.

Then, the first draft of the TEMA-SF was sent to three experts with expertise in both early childhood education and mathematics education for review. Based on the recommendations of the expert reviewers, a second draft of this assessment tool was administered, as a pilot study, to the class of 5-year-old children in Kindergarten P, where the ten embodied mathematics teaching modules had been designed (Chang et al., 2024) for validation and revision purposes with qualitative observations and analyses.

Before administering the pilot study, the research team trained two raters, both of whom were research assistants on the current project and enrolled in the master’s program in the early childhood education department. The inter-rater reliability of the two raters was accordingly analyzed. To assess the inter-rater reliability of the TEMA-SF used in this study, a random subset of the pilot sample (*n* = 10) was independently scored by the two raters who were blind to each other’s scores. An Intraclass Correlation Coefficient (ICC) was calculated using a two-way random-effects model to assess absolute agreement. The results yielded a single-measure ICC of 0.91, with a 95% confidence interval of [0.70, 0.98], *F* (9, 9) = 24.87, *p* < 0.001. According to the benchmarks suggested by Koo and Li (2016), the value indicated a “good” to “excellent” level of reliability, suggesting high objectivity and consistency in the scoring criteria. Furthermore, based on the pilot assessment, the difficulty level was adjusted to align with the children’s capabilities and responses. For example, item 1 was originally designed to assess the core concept “count to 5”, and was revised to assess “count to 20”. As a result, a revised version of this assessment tool, the TEMA-SF (Wu & Chang, 2025), was established to assess young children’s embodied mathematics learning in this quasi-experimental study.

The TEMA-SF includes 19 items to assess young children’s embodied mathematics skills in two major categories: numeracy (12 items) and geometry (7 items). Each item uses a 3-level rubric (10 points per item, totaling 190 points). For most items, the original design is retained with a single task (10 points), while 7 items (i.e., 1~4, 7, 8, 19) have been revised into 2-step items (e.g., 2 tasks; 5 points per step). All items of the TEMA-SF were administered through clinical interviews as the pre- and post-tests to both groups in the Spring semester of 2022. As an example, here are brief descriptions of some items.

1. In the “numeracy” category: (1) For task 1 of item 1, the child needs to count small balls within the basket (20 balls in total). In task 2, the child is asked to divide a large lump of clay into 20 small pieces (similar in size to the small ball), which is particularly sophisticated as it requires dual-task monitoring. That is, the child must coordinate fine-motor control (pinching clay) with mental counting (counting to 20), so that their ability to allocate cognitive resources under physical load (i.e., a hands-on activity) can be assessed. (2) For task 1 of item 4, the child needs to count how many times the instructor claps in a regular pattern. In task 2, the child is asked to count “rhythmic claps (i.e., ‘slow–fast–fast’ rhythm)” to challenge their auditory working memory. Unlike visual counting, where objects serve as spatial markers, auditory counting requires the child to maintain a temporal sequence in their mind without external visual cues. (3) Item 6 requires the child to arrange blocks in rows of five to assess their ability to see “five” as a single entity or unit. (4) Item 11 is a classic test for the “Counting-on from N” strategy (i.e., adding 5 to 7 hidden blocks), indicating a critical milestone where children move beyond counting all to more efficient arithmetic logic.
2. In the “geometry” category: (1) Items 13 and 14 aim to assess the child’s ability to move from naming a shape to describing its differences (e.g., triangles have “three sides”), which marks the transition from the visual level to the descriptive–analytic level of the Van Hiele hierarchy. Item 16 requires the child to differentiate a pentagon from quadrilaterals, which requires higher-level property analysis. (2) The child needs to construct a triangle by using geometric buckle strips in item 15, which involves an understanding of closed shapes and side-length conservation. Successful completion of this task shows that the child understands that the physical properties of the strips must match the geometric requirements of the shape. (3) Item 17 requires the child to first sort a group of different shapes (three types: triangle, square, circle) and then to put them into the sequence as required (i.e., ▲●▲■●). This task requires the child to detect a complex pattern and maintain a repetitive rule in working memory across multiple iterations. (4) For task 1 of item 19, the child needs to form a large triangle from four smaller ones. The child is then asked to take away a triangle from a hexagon and identify the remaining shape with other shapes (i.e., point out that it is like a trapezoid or a rhombus), indicating an advanced ability to perceive embedded geometric figures.

As mentioned, varied manipulatives (e.g., clay, LEGO, geometric buckle strips, small animal models) were used in the TEMA-SF assessment, providing rich embodied experiences that help young children anchor and understand abstract math concepts by demonstrating their capabilities through behavioral actions and multiple representations. Furthermore, different concrete objects or manipulatives were used in some pre-tests and post-tests of the TREMA-SF to reduce the potential impact of the memory effect.

## 3. Results

To answer the two research questions, the findings of this study are presented in two parts: a quantitative analysis of differences in children’s embodied mathematics learning and an exploratory description of clinical interviews within the experimental group.

### 3.1. Analysis of Differences in Children’s Embodied Mathematical Learning

At the beginning of the Spring semester of 2022, all participants (5-year-old children) from the experimental group (*n* = 25 from Kindergarten A) and the control group (*n* = 26 from Kindergarten B) were administered the TEMA-SF as the pre-test to examine the initial status of their mathematics skills before the intervention. After the intervention, at the end of the Spring 2022 semester, the TEMA-SF post-test was administered to assess the final status of both groups. Quantitative comparisons were conducted to examine the differences between the two groups at baseline (before the intervention) and post-intervention. For the experimental group, a within-group comparison was performed to determine whether the intervention, i.e., the implementation of the revised embodied mathematics teaching modules, was effective, indicating developmental growth in the children’s mathematics skills.

#### 3.1.1. Between-Group Baseline Comparison (Pre-Test)

To confirm the initial status of the children’s mathematics skills, an independent *t*-test was conducted to examine differences between the two groups. Descriptive statistics for the TEMA-SF pre-test of these skills are reported in Table 4, including the total score and the two categories (i.e., numeracy and geometry). The mean scores for the experimental and control groups were 158.80 and 157.69, respectively. Initial analysis of this baseline comparison data revealed that there was no significant difference in the total score of the TEMA-SF pre-test between the two groups, *t* (49) = 0.180, *p* > 0.05 (see Table 4 for details). Furthermore, no significant difference was found between the two groups in the “Numeracy” and “Geometry” categories. These results confirm that there was no significant difference in the baseline mathematics skills between the two groups prior to the quasi-experimental intervention.

#### 3.1.2. Between-Group Post-Intervention Analysis

As mentioned above, a revised version of 10 embodied mathematics teaching modules was used in Kindergarten A as the intervention for the experimental group to promote the children’s embodied mathematics learning performance, while the control group continued with the traditional teaching modules and learning activities. At the end of the Spring 2022 semester, a post-test of the TEMA-SF was conducted for post-intervention analysis. The correlation coefficient for the total scores between the pre-test and post-test was 0.66 (*p* < 0.001), indicating a significant positive correlation.

Descriptive statistics for the TEMA-SF post-test of these 5-year-old children’s mathematics skills are presented in Table 5, including the total score and the scores in the two categories (i.e., numeracy and geometry). The mean scores for the experimental and control groups were 172.92 and 159.92, respectively. Follow-up analysis of this post-intervention comparison data showed that significant differences were detected in the total score of the TEMA-SF post-test between the two groups, *t* (49) = 2.446, *p* < 0.05, *η*^2^ = 0.109 (see Table 5 for details). This finding indicates that the young children in the experimental group (Kindergarten A) had significantly superior overall mathematics skills than those in the control group (Kindergarten B). This result reveals that in the experimental group, the intervention was significantly effective in this quasi-experimental study; that is, the designated revised version of 10 embodied mathematics teaching modules effectively enhanced the overall mathematics performance of the children in the experimental group (Kindergarten A) compared to those in the control group (Kindergarten B).

In addition, significant differences were found between the two groups in the “Geometry” category, *t* (49) = 3.057, *p* < 0.01, *η*^2^ = 0.160. This indicates that the children in Kindergarten A had a superior understanding of the “Geometry” concepts after completing the embodied mathematics teaching modules compared to those in Kindergarten B. However, no significant difference was found between the two groups in the “Numeracy” category, although the scores for children in Kindergarten A were slightly higher than for those in Kindergarten B.

#### 3.1.3. Experimental Intervention Implementation and Within-Group Gains

According to the between-group post-intervention comparison, the children in Kindergarten A performed better overall in mathematics and in the geometry category. These results demonstrate the effectiveness of the revised version of 10 embodied mathematics teaching modules, indicating that these modules promoted the children’s overall understanding of mathematical concepts. In Table 6, analysis of the experimental group’s internal progress showed a significant increase in the TEMA-SF from a pre-test mean of 158.80 to a post-test mean of 172.92 after the intervention. A paired-samples *t*-test confirmed that this gain was statistically significant, *t* (24) = −3.965, *p* < 0.001, *η*^2^ = 0.396. Significant improvements were also found across the “Numeracy” (e.g., verbal counting, object counting, number comparison, and compositions/decompositions) and “Geometry” (e.g., shape recognition, composition of shapes, and spatial orientation) categories. These results demonstrate that the implementation of embodied mathematics teaching modules was significantly beneficial for the acquisition of various mathematical concepts.

Conversely, the control group’s mean scores (pre-test: 157.69; post-test: 159.92) showed no significant changes regarding overall score and across the two categories, as assessed by paired-samples *t*-tests (see Table 7 for details). This suggests that the conventional teaching model in Kindergarten B did not yield significant growth in the mathematics skills during the study period.

### 3.2. Exploratory Description of Clinical Interviews Within the Experimental Group

Based on the quantitative results of the pre- and post-test scores on the TEMA-SF, the 25 5-year-old children in Kindergarten A showed greater improvement in mathematics skills after the intervention. As indicated above, the TEMA-SF was administered through clinical interviews to observe the targeted children’s behavioral actions (e.g., verbal expression and physical movement). The use of clinical interviews in the assessment process provided deeper insight into young children’s mathematical learning performance, which was analyzed in depth through textual description. The findings were presented in the following two categories:

1 “Numeracy”

First, these children demonstrated solid basic counting skills but demonstrated room for development in numerical conservation, mental arithmetic, memory, and high-order number concepts. In auditory counting tasks up to 10, they could accurately match the number of claps to a stable rhythm, indicating mastery of one-to-one correspondence and stable-order principles. However, when dividing a large lump of clay into 20 small pieces, they often focused on the psychomotor action instead of on simultaneous counting, resulting in more than 20 pieces (in a few cases). This result revealed that the integration of operational actions with counting processes and self-monitoring abilities is still ongoing. In grouping activities within 20, they arranged objects into sets of five as instructed and verified the total by counting individually. Some of them benefitted from a skip-counting-by-five strategy during the post-test, which possibly reflects emerging concepts of grouping and multiplicative thinking. In short, their counting strategies showed a transition from one-by-one counting to more structured approaches.

Secondly, the children accurately grouped small-animal models by category, without being influenced by color in classification and memory tasks, demonstrating stable categorical concept development. Nevertheless, when concrete objects or manipulatives were removed, they struggled to recall the total number, suggesting that their numerical representations still depend on concrete support. Therefore, further development of abstract numerical representation and integration with working memory is necessary. Moreover, in tasks involving combinations to make 10, most children completed basic addition, but confusion arose when the base number exceeded four. This finding may indicate that automaticity in the decomposition and composition of 10 has not yet been achieved, and that their concept of number complements is still developing. When comparing two-digit numbers (e.g., 27 vs. 32; 20 vs. 30) and single-digit numbers, they correctly judged relative quantities. This result shows a good understanding of place value, likely due to frequent counting practices in the kindergarten setting.

Overall, these young children demonstrated stable foundational counting abilities and an emerging understanding of place value, as well as developing grouping and strategic counting skills. However, they remained in a transitional stage regarding numerical conservation, the mental representation of quantities, and the decomposition and composition of numbers beyond 10. In fact, this developmental model reflects the typical process of kindergarten children from concrete operational experiences to more abstract numerical thinking.

2 “Geometry”

These children’s geometric abilities demonstrated a developmental profile characterized by mature shape recognition, whereas skills in geometric feature analysis and spatial transformation were still under development. In basic shape identification tasks, most children correctly named commonly known shapes, including triangles, squares, rectangles, and circles, indicating a strong foundation in shape naming. However, when comparing various shapes, only a few children could explicitly reference geometric attributes such as the number of sides, angles, or side-length relationships. Many children still relied on perceptual descriptions, such as using the word “pointy,” and did not recognize that squares and rectangles have angles as well. These results suggest that their understanding of shapes was primarily based on holistic visual recognition rather than analytical consideration of geometric attributes.

Moreover, regarding shape construction tasks, some children demonstrated emerging geometric structural awareness by considering the number of sides and angles, vertex connections, and side-length correspondences, for example, by recognizing that opposite sides of a rectangle are equal. In contrast, others combined multiple connecting strips into a single side without verifying the consistency of the side length, resulting in distorted shapes. Based on these behavioral observations, their understanding of side-length conservation and entire shape constraints had not yet fully developed. In addition, skills in recognizing and constructing rhombi and trapezoids were weaker than for more familiar shapes, which may result from limited experience with non-prototypical shapes. In addition, in pattern-replication tasks, most children accurately reproduced a single sequence, demonstrating basic pattern recognition, and only a few children extended the sequence beyond one unit.

Further, in part-to-whole construction tasks, most children were more successful at forming horizontal rectangles than vertical ones, indicating that their mental rotation and spatial transformation abilities were not yet fully developed. In shape decomposition and reconstruction tasks, approximately half of the children successfully combined four small triangles into a larger triangle and identified a similar resulting shape after removing one small triangle piece. This outcome reflects an emerging understanding of part–whole relationships and shape conservation. However, most children were unable to properly describe geometric properties such as side length, angle, and area for trapezoids and rhombi, which may indicate the need for more exploratory experiences related to real-life situations.

In summary, these young children demonstrated stable shape recognition and basic classification abilities, as well as emerging skills in construction and pattern understanding. However, they remained in a transitional stage with respect to geometric attribute analysis, mental rotation, reasoning concerning side–angle relationships, and specific recognition of non-prototypical shapes. This developmental pattern reflects young children’s typical progression from intuitive visual recognition to attribute-based geometric thinking.

Aside from the exploratory descriptions of the two categories, it was noteworthy that, in addition to learning through the revised 10 embodied mathematics teaching modules, young children in the experimental group also had access to mathematics learning centers in the classroom. This provided beneficial opportunities for daily free exploration and hands-on manipulation activities, which aligned with the implementation of the revised modules. These supplementary learning opportunities were provided in several modes. (1) Number and quantity: small animal models were provided for sorting and sequencing; playing cards were provided for bridging abstract symbols and quantities; and play money was provided for multi-digit addition and decimal conversion. (2) Geometry and shape: materials ranged from 2D pattern blocks and tangrams to 3D magnetic tiles, vertex beads, and modeling rods, allowing for both guided imitation and open-ended exploration. (3) Spatial cognition: 2D puzzles, 5 × 5 geoboards with pattern cards, and board games focusing on directional paths and 3D spatial design (e.g., Tetris-style blocks) were provided. (4) Temporal concepts: classrooms were equipped with custom clocks with movable hands and scales representing 1–12 h and 1–60 s. These learning materials and manipulatives were essential in building these young children’s understanding of the targeted mathematical concepts by providing diverse embodied experiences that enhanced their “perception-for-action” capabilities.

## 4. Discussions

The following discussion, grounded in the two major contributions of this study, is presented in two parts. First, based on the effective intervention in the experimental group, the embodied mathematics teaching modules can be used in both in-service and pre-service teacher professional development. They help early childhood educators rethink the embodied approach for accelerating young children’s mathematical power. Secondly, the TEMA-SF can serve as a comprehensive yet developmentally appropriate assessment tool for measuring young children’s mathematics learning performance. It also highlights the importance of contextualizing assessment tools for local educational settings, providing both quantitative and exploratory insights into young children’s embodied mathematical learning. In addition, a subsection on the limitations of this study and suggestions for future studies was proposed.

### 4.1. Rethinking the Embodied Approach for Accelerating Young Children’s Mathematical Power

The rich assortment of concrete manipulatives and real-world tasks provided multiple entry points for young children’s engagement, further supporting embodied approaches that leverage multimodal experiences for mathematical reasoning (Barsalou, 2008; Tran et al., 2017). Additionally, in this study, a significant impact of the embodied mathematics teaching modules on young children’s mathematical power, particularly in numeracy and geometry, was observed. In the experimental group, the young children demonstrated notable growth in conceptual understanding, procedural fluency, spatial reasoning, and problem-solving strategies after participating in the embodied learning activities, as evidenced by the TEMA-SF post-test results (Wu & Chang, 2025). Moreover, the results highlighted that the children’s mathematical power extended beyond simply producing correct answers to encompass cognitive flexibility, strategic thinking, and problem-solving in real-life contexts. For instance, when planning routes to measure distances, children coordinated physical actions (using “foot rulers”) with mathematical reasoning, illustrating an integrated perception–action cycle that supports embodied cognition and promotes deeper understanding (Abrahamson & Shulman, 2019; Tran et al., 2017). This demonstrates that young children’s mathematical power is not only about procedural skills but also about actively constructing mathematical meaning through authentic behavioral interaction with the environment and manipulatives. Therefore, the professional development process, which ensures adequate prerequisite knowledge and capabilities, played a valuable role in preparing Kindergarten A teachers to design and implement the embodied mathematics teaching modules, in turn enhancing their children’s mathematics learning performance.

Several scholars have argued that all mathematical learning is grounded in an embodied understanding of the world (Abrahamson, 2017; Abrahamson & Sánchez-García, 2016; Hutto et al., 2015), which emphasizes the need to provide children with learning opportunities through sensorimotor experiences and bodily interactions with the designated environment (Glenberg, 2010). During the embodied mathematics learning process, the children in Kindergarten A learned mathematical concepts by acting, manipulating concrete objects, and exploring real-life situations. For example, they measured distances using their own bodies, assembled unit blocks, and designed patterns with manipulatives. This kind of embodied mathematics learning context, which echoes the embodied cognition theory (Barsalou, 2008; Wilson, 2002) and the “embodied design” approach (Abrahamson, 2009, 2014; Abrahamson & Shulman, 2019), allowed the children to learn in a “perception–action cycle” (Tran et al., 2017). That is, through hands-on experience and bodily movements, these young children experienced changes in conceptual understandings that, in turn, influenced later learning in a cyclical process. These embodied “perception-for-action” interactions among young children’s perceptions, their behavioral actions, and the learning environment are beneficial for the developmental change in their mathematics learning performance (Chang et al., 2024) and also bridge concrete experience with abstract mathematical thinking to promote their conceptual understanding (Alibali & Nathan, 2012; Sarama & Clements, 2009).

Even though these children showed superior gains in embodied mathematics skills, there was still room for improvement in both the “numeracy” and “geometry” categories. For the numeracy category, the findings showed that they were in a transitional stage regarding numerical conservation, the mental representation of quantities, and the decomposition and composition of numbers beyond 10. This shows that they were in the process of moving from concrete operational experiences to more abstract numerical thinking. Regarding the geometry category, further support was needed to promote their skills in geometric attribute analysis, mental rotation, reasoning about side–angle relationships, and specific recognition of non-prototypical shapes. This developmental pattern showed they were transitioning from intuitive visual recognition to attribute-based geometric thinking. This indicates that if we can provide children more opportunities to progress “from doing to thinking-about-doing” (Abrahamson, 2024, p. 149) through an “embodied design” approach (Abrahamson, 2009, 2014; Abrahamson & Shulman, 2019), it will be beneficial for teachers assisting with the children’s learning and, in turn, enhance their mathematical understanding and skills.

Furthermore, young children in the experimental group had superior growth in overall scores and the “Geometry” category in the between-group post-intervention comparison; however, no significant difference was found between the two groups in the “Numeracy” category. In fact, this result reflected a common phenomenon in Taiwanese educational settings. Based on findings from several studies conducted in Taiwan, early mathematical competence is commonly conceptualized with a strong emphasis on “number and quantity” as foundational components while learning. It was evident that preschool children already possess basic numerical abilities prior to formal instruction. These abilities can be significantly enhanced through traditional learning methods such as counting and arithmetic (Lai et al., 2022). Most research in mathematics education for young children focuses on numerical competence; for example, Lin (2023) used number lines to support the development of number sense and basic arithmetic skills. Developmental review studies also indicated that children’s number concepts develop progressively with age and exhibit domain-specific characteristics, which continue to be actively investigated in the literature (Chiu & Ko, 2015). Consequently, how embodied mathematics teaching can promote young children’s numeracy performance requires further, long-term, or qualitative research to explore and confirm its effectiveness.

### 4.2. TEMA-SF Can Serve as a Comprehensive Yet Developmentally Appropriate Assessment Tool for Measuring Young Children’s Mathematics Learning Performance

The TEMA-SF’s alignment with the theoretical framework of embodied cognition enabled the assessment not only of the children’s final answers but also of their reasoning processes, problem-solving strategies, and embodied interactions with materials. This approach is consistent with previous research emphasizing that young children’s mathematical understanding is best captured through dynamic, process-oriented assessments rather than traditional static testing (Alibali & Nathan, 2012; Clements & Sarama, 2021; Sarama & Clements, 2009). The use of clinical interviews to administer the TEMA-SF further strengthened its validity in this context. By observing children’s behavioral actions, including verbal explanations, gestures, and physical interactions with manipulatives, we were able to assess both conceptual understanding and embodied engagement, which aligns with the principles of “perception-for-action” in embodied mathematics learning (Abrahamson & Lindgren, 2014; Glenberg, 2010). For instance, learning tasks that combined counting with manipulating objects or forming geometric shapes allowed for the simultaneous assessment of cognitive and sensorimotor coordination.

However, while the TEMA-SF effectively measured developmental progress in both numeracy and geometry, numeracy gains were less pronounced than those for geometry in this study cohort. This may reflect the context-specific strengths of embodied assessment, which more directly capture spatial and geometric reasoning but may require additional adaptation or scaffolding to fully assess abstract numerical operations (Boaler, 2016; Van de Walle et al., 2022). The careful cultural adaptation of the TEMA-SF, through translation, the addition of clear instructions and concrete objects, and expert reviews and pilot testing, was essential to ensure reliability and appropriateness for Taiwanese kindergartners, highlighting the importance of contextualizing assessment tools for local educational settings (Chang et al., 2024; Wu & Chang, 2025). In summary, the TEMA-SF proved to be a robust, theory-aligned, and process-sensitive assessment instrument, providing both quantitative and exploratory insights into young children’s embodied mathematical learning performance. Its implementation also demonstrates that assessments in early mathematics education can move beyond simply recording correct answers to recognizing developmental trajectories, embodied reasoning, and conceptual growth (Barsalou, 2008; Clements & Sarama, 2021; Sarama & Clements, 2009).

### 4.3. Limitations and Future Studies

#### 4.3.1. Limitations

As noted in previous sections, the revised version of the 10 embodied mathematics teaching modules, as the intervention for the experimental group, significantly promoted children’s embodied mathematics learning, as evidenced by their scores overall and in the geometry category. In addition, the embodied assessment tool, TEMA-SF, can serve as a comprehensive yet developmentally appropriate measure of young children’s mathematics learning performance. However, this quasi-experimental study had several limitations. (1) Sample size and diversity: The scope of this study was limited to a quasi-experimental design contextually involving 51 children from two kindergartens. This small sample size and localized setting may limit the generalizability of the findings to different age groups, socio-economic backgrounds, or larger, more diverse populations. (2) Intervention with the embodied mathematics teaching modules: The quantitative findings focus on “post-test” gains, along with the exploratory descriptions after the intervention. The intervention used in the experimental group, namely the revised embodied teaching modules, requires necessary modifications and adaptations to align with and be merged into each kindergarten’s original curriculum and instructional plans. (3) Teachers’ PD: The design and implementation of the embodied modules were successful in Kindergarten A. Teachers who want to use this embodied teaching approach need appropriate professional development to effectively understand and implement the principles and practices of embodied mathematics teaching and learning. (4) Embodied mathematics assessment: Before assessing young children’s embodied mathematics learning performance, raters of the TEMA-SF must receive appropriate training and establish inter-rater reliability before administering the assessment.

#### 4.3.2. Future Studies

Based on the findings and discussions, three academic and practical implications emerged. First, the use of embodied mathematics teaching modules, along with multimodal learning, is beneficial for young children’s mathematics learning performance. To further verify the effectiveness of this embodied approach, future research will require larger sample sizes for quantitative comparisons and long-term follow-up for qualitative analyses. In addition, since the current study focused solely on quantitatively examining the effectiveness of the embodied modules through a quasi-experimental design, the qualitative process analysis of how these modules were implemented in the classroom was not included in this report. These qualitative results may be published in a separate paper in the future. It is also recommended that future research focus on qualitative studies that explore how kindergarten teachers use embodied mathematics teaching modules to promote their young children’s mathematics learning, with an emphasis on interviews with teachers and students, as well as classroom observations of the multi-modal teaching and learning processes.

Secondly, the design and implementation of the embodied mathematics teaching modules and the TEMA-SF assessment were based on the execution of three research projects since 2018. Teachers’ PD formed the foundation of previous research findings (Chang et al., 2024; Wu & Chang, 2022) and the current study (Wu & Chang, 2025). Future studies may draw upon the researchers’ previous work (e.g., Chang et al., 2024) on teachers’ PD, including a sustainable “task design PD model,” to plan similar embodied research projects in different practical settings. Moreover, the embodied assessment tool, the TEMA-SF, can be used to conduct future research on how to assess young children’s embodied mathematics learning performance, both quantitatively and qualitatively. Future studies can also focus on developing similar or different embodied assessment tools to improve the quality of teaching and learning in early childhood education.

## 5. Conclusions

### 5.1. There Are Statistically Significant Differences in the Mathematics Learning Gains Between the Two Groups of Young Children After the Intervention

Based on the quantitative findings, both groups began with similar baseline (pre-test) scores; however, the experimental group’s post-test “overall” scores and “geometry” scores were significantly higher than those of the control group. These findings indicated that, compared to those children in Kindergarten B, the design and implementation of the revised version of the 10 embodied mathematics teaching modules significantly improved the targeted young children’s mathematics learning performance in Kindergarten A. In addition, a within-group comparison in the experimental group confirmed that the intervention, i.e., the revised embodied mathematics teaching modules, was significantly effective, indicating developmental growth in the children’s embodied mathematics skills. The 25 5-year-old children in Kindergarten A showed superior improvement in both the “numeracy” and “geometry” categories.

### 5.2. Exploratory Description of Clinical Interviews Showed Improvements in Children’s Mathematics Performance Within the Experimental Group

Based on the exploratory descriptions of clinical interviews of the TEMA-SF in the experimental group, it was found that the revised embodied mathematics teaching modules provided an enriched learning environment, associated with the learning centers and various kinds of manipulatives and teaching aids, in which these young children could reflect on how their body movements and hands-on experiences relate to the specific mathematical concepts they were learning. This type of multimodal learning, recognizing the importance of visual, auditory, kinesthetic, and tactile modalities in the learning process, helped these children engage with mathematical concepts and build deeper mathematical understanding by grounding abstract concepts in physical activity. Echoing the quantitative results, the exploratory descriptions of clinical interviews also revealed that children in Kindergarten A, who engaged in multimodal, manipulative-rich learning activities, exhibited a deeper geometric understanding and strategic counting behaviors. Consequently, the findings of this study were consistent with the embodied cognition perspective that mathematical learning is grounded in behavioral interactions and perception-for-action cycles. Furthermore, the development of the TEMA-SF provides a contextually and developmentally appropriate assessment tool for assessing young children’s embodied mathematics learning performance.

## Figures and Tables

**Table 1:** Modules’ concepts aligned with the Common Core State Standards for Mathematics.

CCSS	1	2	3	4	5	6	7	8	9	10
Counting and Cardinality			^V^	^V^		^V^		^V^	^V^	^V^
Operations and Algebraic Thinking				^V^		^V^		^V^		
Number and Operations in Base Ten				^V^				^V^		
Geometry	^V^	^V^			^V^		^V^		^V^	
Measurement and Data						^V^		^V^		^V^

^V^ indicates the module’s learning concept is consistent with the Common Core State Standards for Mathematics (CCSSI, 2023).

**Table 2:** Modules’ learning activities aligned with NCTM process standards.

NCTM	1	2	3	4	5	6	7	8	9	10
Problem-Solving	^V^	^V^	^V^	^V^		^V^		^V^	^V^	^V^
Reasoning and Proof	^V^	^V^	^V^	^V^	^V^	^V^	^V^	^V^	^V^	^V^
Communication		^V^	^V^					^V^		^V^
Connections			^V^	^V^		^V^		^V^	^V^	^V^
Representation	^V^	^V^	^V^	^V^	^V^	^V^	^V^	^V^	^V^	^V^

^V^ indicates the module’s learning concept is consistent with NCTM (2000) standards.

**Table 3:** Module 10—original and revised plans.

Category	Original Module	Revised Module
Mathematical Concepts	Number, quantity, shape, space, measurement	
Learning Content	Counting numbers, units of measurement, informal measurement, and formal measurement	
Teaching Resources	Balloons, kite strings (made of different materials), a camera, and poster papers	Cloth ruler and plastic rope, children’s hands and feet, unit blocks, and poster papers
Target for Measurement	Height of the mama tree (outside of the classroom, free play zone)	Distance to teacher’s office (on the same floor as the children’s classroom)

**Table 4:** Descriptive statistics and independent *t*-test results for the TEMA-SF pre-test.

Category	Group	*n*	*M*	*SD*	*t*	*p*
Numeracy	Experimental—Kindergarten A	25	103.40	10.45	0.484 ^n.s.^	0.631
	Control—Kindergarten B	26	101.62	15.32		
Geometry	Experimental—Kindergarten A	25	55.40	11.05	−0.212 ^n.s.^	0.833
	Control—Kindergarten B	26	56.08	11.74		
Total Score	Experimental—Kindergarten A	25	158.80	18.84	0.180 ^n.s.^	0.858
	Control—Kindergarten B	26	157.69	24.51		

^n.s.^ *p* > 0.05.

**Table 5:** Descriptive statistics and independent *t*-test results for the TEMA-SF post-test.

Category	Group	*n*	*M*	*SD*	*t*	*p*
Numeracy	Experimental—Kindergarten A	25	108.60	10.96	1.531 ^n.s.^	0.132
	Control—Kindergarten B	26	103.19	14.02		
Geometry	Experimental—Kindergarten A	25	64.32	6.96	3.057 **	0.004
	Control—Kindergarten B	26	56.73	10.37		
Total Score	Experimental—Kindergarten A	25	172.92	14.09	2.446 *	0.018
	Control—Kindergarten B	26	159.92	22.69		

* *p* < 0.05; ** *p* < 0.01; ^n.s.^ *p* > 0.05.

**Table 6:** Descriptive statistics and paired-samples *t*-test results of the experimental group.

Category	Assessment	*n*	*M*	*SD*	*t*	*p*
Numeracy	Pre-test	25	103.40	10.45	−2.205 *	0.037
	Post-test	25	108.60	10.96		
Geometry	Pre-test	25	55.40	11.05	−4.188 ***	0.000
	Post-test	25	64.32	6.96		
Total Score	Pre-test	25	158.80	18.84	−3.965 **	0.001
	Post-test	25	172.92	14.09		

* *p* < 0.05; ** *p* < 0.01; *** *p* < 0.001.

**Table 7:** Descriptive statistics and paired-samples *t*-test results of the control group.

Category	Assessment	*n*	*M*	*SD*	*t*	*p*
Numeracy	Pre-test	26	101.62	15.32	−0.767 ^n.s.^	0.450
	Post-test	26	103.19	14.02		
Geometry	Pre-test	26	56.08	11.74	−0.357 ^n.s.^	0.724
	Post-test	26	56.73	10.37		
Total Score	Pre-test	26	157.69	24.51	−0.763 ^n.s.^	0.453
	Post-test	26	159.92	22.69		

^n.s.^ *p* > 0.05.

## Data Availability

As mentioned in the text, the quantitative data in this study primarily came from administering the TEMA-SF via clinical interviews (including video recordings of young children’s learning performance). In addition, the qualitative data also included video recordings of classroom observations and other related data. As a result, the data presented in this study are available upon request from the corresponding author due to privacy and ethical reasons.

## Abbreviations

The following abbreviations are used in this manuscript:

REMA	Research-Based Early Mathematics Assessment
REMA-SF	Research-Based Early Mathematics Assessment—Short Form
TEMA-SF	Taiwanese Embodied Mathematics Assessment—Short Form
PD	Professional Development
